# 

**Published:** 1886-01

**Authors:** 


| | PAGE |
|---|---|
| ORIGINAL COMMUNICATIONS.—The Comparative Anatomy of the Pyramid Tract. E. C. Spitzka, M.D. | 1 |
| Tuberculosis, from Anatomical, Etiological, and Preventive Standpoints, with especial reference to Phthisis Pulmonum. Frank S. Billings, V.S. | 62 |
| Milk: From a Medico-Sanitary Standpoint. Thomas B. Rogers, D.V.S. | 101 |
| Azoturia, more especially with reference to its Nomenclature and Pathology. Richard W. Burke, M.R.C.V.S. | 139 |
| George Fleming, LL.D., F.R.C.V.S., Principal Veterinary Surgeon to the British Army | 143 |
| EDITORIAL DEPARTMENT | 147 |
| PROCEEDINGS VETERINARY MEDICAL SOCIETIES | 151 |
| OBITUARY | 158 |
| REVIEWS | 159 |

